# Navigating the shadows: the impact of mindfulness, cognitive fusion, and coping strategies on psychological distress among mental health workers in Timor Leste

**DOI:** 10.1007/s44192-025-00253-y

**Published:** 2025-08-07

**Authors:** Gaspar Quintao, Noviyanti Carla Tilman Leite, Nicholas Tze Ping Pang, Eugene Boon Yau Koh, Jhia Mae Woo, Marina Abdul Rahman Sabri, Kah Mun Wan, Noor Melissa Noor Hadi, Ming Gui Tan, Assis Kamu, Chong Mun Ho

**Affiliations:** 1 Guido Valdarez National Hospital, Dili, Timor-Leste; 2 Pradet Timor Leste, Dili, Timor-Leste; 3 Faculty of Medicine and Health Sciences, Universiti Malaysia Sabah, Jalan UMS, 88400 Kota Kinabalu, Sabah Malaysia; 4 University Putra Malaysia, Kuala Lumpur, Malaysia; 5 Sunway University, Bandar Sunway, Selangor Malaysia; 6 Hospital Sultanah Aminah, Johor Bahru, Malaysia; 7 ACT Kuala Lumpur, Kuala Lumpur, Malaysia; 8 University Malaysia Sabah, Kota Kinabalu, Malaysia

**Keywords:** Mindfulness, Cognitive fusion, Coping strategies, Psychological distress, Mental health workers, Timor Leste, Vicarious trauma, Compassion fatigue, Secondary traumatic stress disorder

## Abstract

**Background:**

Mental health workers in post-conflict settings such as Timor Leste face distinct stressors stemming from limited human resources, underdeveloped systems, and ongoing socio-political instability, all of which increase the risk of psychological distress among these professionals. Consequently, constructs such as mindfulness, cognitive fusion, and coping strategies are essential not only theoretically significant, but also serve as practical targets for strengthening mental resilience of these professionals in these high-burden environments. This study aims to investigate the relationships between mindfulness, cognitive fusion, coping strategies, and psychological distress (depression, anxiety, and stress) among mental health workers in Timor Leste.

**Methods:**

A cross-sectional study design was employed, involving a convenience sample of 37 mental health workers from PRADET and the national referral hospital in Dili. Mindfulness was assessed using the Toronto Mindfulness Questionnaire (TMQ), psychological flexibility using the Acceptance and Action Questionnaire (AAQ-II), cognitive fusion was measured using the Cognitive Fusion Questionnaire (CFQ), and coping strategies were evaluated using the DBT-Ways of Coping Checklist (DBT-WCCL). Depression, anxiety, and stress were measured using the Depression Anxiety Stress Scales (DASS-21). All scales were using English validated versions. Descriptive statistics, Pearson correlation coefficients, and multiple regression analyses were used to analyze the data.

**Results:**

Significant positive correlations were found between Depression and Anxiety (Spearman’s rho = 0.649, *p* < 0.001), and between Depression and Stress (Spearman’s rho = 0.753, *p* < 0.001). Depression was also significantly correlated with Cognitive Fusion (Spearman’s rho = 0.445,* p* = 0.006) and Blaming Others (Spearman’s rho = 0.422, *p* = 0.009), and negatively correlated with Coping Strategies (Skills Use) (Spearman’s rho =– 0.341, *p* = 0.039). Anxiety and Stress were highly correlated (Spearman’s rho = 0.855, *p* < 0.001), and both were significantly associated with Cognitive Fusion, General Dysfunctional Coping, and Blaming Others. Mindfulness (De-Centering) showed a strong positive correlation with Mindfulness (Curiosity) (Spearman’s rho = 0.770, *p* < 0.001), and was also weakly associated with General Dysfunctional Coping (Spearman’s rho = 0.343, *p* = 0.038). Overall, the results suggest that higher levels of depression, anxiety, and stress are linked to greater cognitive fusion and dysfunctional coping, while effective coping skills are negatively associated with depression.

**Conclusion:**

The findings highlight the critical roles of cognitive fusion and coping strategies in predicting psychological distress among mental health workers in Timor Leste. Cognitive fusion and dysfunctional coping strategies were associated with higher levels of depression, anxiety, and stress. Adaptive coping strategies, such as skills use, were linked to lower levels of depression. Given the high risk of vicarious trauma, compassion fatigue, and secondary traumatic stress disorder in this population, targeted interventions promoting mindfulness and adaptive coping skills are essential. Addressing these factors can enhance resilience and well-being among mental health professionals, ultimately improving the quality of care provided to their clients.

## Background

Timor Leste, a Southeast Asian nation, has endured a tumultuous history marked by colonialism, occupation, and a prolonged struggle for independence. The country achieved sovereignty in 2002 after years of violent conflict and political upheaval, leaving a significant portion of its population with deep psychological scars. This historical context has contributed to a high prevalence of mental health issues, including depression, anxiety, and stress among its citizens, particularly those involved in mental health professions.

Mental health workers in Timor Leste, who are often at the forefront of addressing the mental health needs of the population, face unique challenges. They work in an environment with limited resources and support, compounded by their own potential experiences of trauma associated with the after-effects of the shared conflicts. There is no research data documenting the number of mental health workers in Timor Leste as this research has yet to be done; however, from personal interviews with the only local mental health workers, there is only one psychiatrist and one clinical psychologist in public health services. The social work and counselling is hence performed by the Psychosocial Recovery and Development in East Timor (PRADET), the voluntary sector in Timor Leste, of which there were 28 individuals in 2024. Mental health workers in Timor Leste primarily consist of social workers and peer counsellors, who are informally trained in psychological work, as there is only one psychiatrist covering the whole nation and no clinical psychologists currently in the state healthcare service as of 2024 in Timor Leste. There is only one trained counsellor in PRADET who only works in the voluntary sector, but does not serve the general population. Both trained workers were not involved as respondents but as coordinators in this study.

Understanding the mental health of these professionals is crucial, as it directly impacts their well-being and their capacity to provide effective care. This study aims to examine the relationship between mindfulness, cognitive fusion, coping strategies, and psychological distress (depression, anxiety, and stress) among mental health workers in Timor Leste.

## Literature review

Timor Leste's history of conflict and trauma has left a lasting impact on the mental health of its population. Research indicates populations exposed to prolonged violence, warfare and other forms of violent political upheaval and instability are at risk of manifesting extreme forms of maladaptive cognition and coping skills in the post-conflict period [26]. The mental health workforce in Timor Leste, comprising professionals from organizations like PRADET and the national referral hospital in Dili, is tasked with addressing these widespread issues. However, the mental health of these workers themselves is an area of concern, as they may also suffer from the effects of their country's traumatic history.

Mental health workers in post-conflict settings such as Timor Leste operate under unique stressors that differentiate their experiences from those in more stable environments. These professionals are often exposed to secondary trauma through their work with severely affected clients, while simultaneously grappling with unresolved personal and collective trauma stemming from the nation's conflict-ridden past. Studies from other post-conflict regions, such as Rwanda and Bosnia, have shown that mental health workers frequently experience burnout, compassion fatigue, and symptoms of post-traumatic stress disorder (PTSD) due to the ongoing demands of their roles and limited psychosocial support structures [5, 28].

In Timor Leste, mental health challenges are compounded by limited human resources, systemic underdevelopment, and socio-political instability, all of which may exacerbate stress, anxiety, and depressive symptoms among professionals. Therefore, variables such as mindfulness, cognitive fusion, and coping strategies are critical not just as psychological constructs, but as practical targets for enhancing the mental resilience of workers operating in these high-burden environments.

Research further underscores that psychological flexibility and mindfulness-based practices may be especially relevant and culturally adaptable in collectivist, post-conflict societies like Timor Leste. Mindfulness-based interventions have been effectively implemented among mental health professionals in high-stress settings, demonstrating reductions in emotional exhaustion, depression, and anxiety [1, 22]. 

Similarly, maladaptive coping strategies—particularly those centered around avoidance or externalizing blame—are linked to increased psychological distress in emergency workers exposed to dehumanising trauma [30] (Kirby et al., 2011). The cultural values prevalent in Timorese society, such as deference to communal norms, spiritual explanations for suffering, and collective responsibility, may shape how individuals interpret distress and respond to it. These cultural lenses could influence tendencies toward cognitive fusion, particularly in the form of fused beliefs about personal worth, fate, or intergenerational suffering.

Maladaptive coping strategies, especially avoidance, are both shaped by culture and circumstances uniquely in Timor Leste. Due to the multiple years of genocide and trauma experienced in Timor Leste, where every extended family suffered loss of life, major morbidity or permanent disability, there is a collective experience of either PTSD for post-traumatic stress symptoms [29]. Hence, as part of the symptom profile, avoidance is actually an understandable and potentially even adaptive defence mechanism to block out the most traumatic memories experienced during the conflict, to prevent re-emergence and re-experiencing of trauma. However, in the case of commonplace psychological symptoms, avoidance then alters its function to become maladaptive.

Culturally as well, Timorese culture is centred on prioritising the interest and consensus of the majority over individual desire. Hence, avoidance is frequently the best way to suppress individual will. In addition, there is major importance attached to divine explanations and “supernatural” causations for suffering as a manifestation of the will of God [3]. Hence to resolve the cognitive dissonance created by the monumental amount of suffering Timor Leste has experienced, a great number of mental avoidance and disengagement strategies would have to be employed to synchronise external reality and internal cognitions. As such, understanding and addressing cognitive fusion and coping strategies through contextually sensitive mindfulness-based approaches can help promote sustainable well-being among mental health workers in Timor Leste.

Persistent feelings and “fused” beliefs of injustice related to human rights violations might be an important factor leading to mental distress such as depression and post-traumatic symptoms in post-conflict populations [2]. Cognitive fusion, a concept from Acceptance and Commitment Therapy (ACT), refers to the tendency to take thoughts literally and get entangled in them, which can exacerbate psychological distress [11, 12]. High levels of cognitive fusion are associated with increased symptoms of depression and anxiety [7, 8] as well as post-traumatic stress [14]. The mediation of cognitive fused on individuals’ post-traumatic cognition often heightens individuals’ perceptions of current or future threats and diminishes perceptions of personal competence and value (i.e. self-blame and sense of hopelessness) or judgement of external factors (i.e. blaming and distrust towards others), further exacerbating emotional distress [2, 6].

The counterprocess of cognitive fusion is cognitive defusion. Cognitive defusion serves as a mechanism of mindfulness that views thoughts as temporary mental events that need not influence behaviour. Mindfulness, defined as the awareness that emerges through paying attention on purpose, in the present moment, and non-judgmentally to the unfolding of experience moment by moment [15], has been shown to have a protective effect against psychological distress. Studies have found that mindfulness can reduce symptoms of depression and anxiety by promoting emotional regulation and reducing rumination [13, 14]. Theoretical explanations unpinning the effectiveness of mindfulness suggest that the act of purposefully attending with openness and non-judgmentally leads to a shift in individuals’ perspectives of the events from accepting them literally as truths to embracing them as simply just thoughts. For example, trauma-related stimuli can easily produce intrusive thoughts caused by confusion about the content of those thoughts as real or happening in reality without being aware that they are simply thoughts as-is. Mindfulness-driven defusion may facilitate the ability to view memories related to traumatic experiences as transient memories rather than currently threatening events and less automatic maladaptive responses to trauma cues [2, 14]. Hence mindfulness is predicted to increase mindful attention awareness and reduce cognitive fusion to enable people to recognise unpleasant emotions or memories in a non-judgmental manner.

The identification of adaptive and maladaptive coping strategies is often the target of research inquiry due to their evident role in the population’s emotional and mental health following stressful life events, including collective traumatic events [27] (Littleton et al., 2017). According to Lazarus and Folkman [17], coping, conceived as a process, refers to individuals' cognitive and behavioural efforts to master, reduce or allow the internal and external demands of a stressful encounter. Strategies focused on solving problems such as planning how to resolve the stressor by seeking information about the stressor or actively attending to one’s emotions using mindfulness and acceptance [21] are shown to be effective adaptive coping strategies. Maladaptive coping strategies include those focused on disengaging or withdrawing from internal and external experiences (avoidance) or in opposition to the spectrum, overidentifying with the internal and external experiences as their literal truths (fusion). Adaptive coping strategies can mitigate the impact of stress and trauma, while maladaptive strategies can exacerbate it [17].

Given the unique historical and cultural context of Timor Leste, this study aims to explore the interplay between mindfulness, cognitive fusion, coping strategies, and psychological distress among mental health workers in the country. Understanding these relationships can inform interventions to support the mental health of these essential professionals.

## Methods

### Ethics approval

Ethical approval was obtained from the Universiti Malaysia Sabah Medical Research Ethics Committee (Approval Code: JKEtika 4/24 (3)).

### Participants

The study sample consisted of thirty-seven mental health workers from PRADET, the national mental health non-govermental organisation (NGO) in Timor Leste, and all mental health workers in the main referral hospital in Dili, Timor Leste. All the mental health workers had at least a high school diploma and had years of practical experience in their field, but all of them have not received any formalised graduate level courses in counselling skills, psychology, or mental health. All of them have only attended ad hoc or informal training on the job, just like the one we were providing.

A convenience sampling method was used to recruit participants. Inclusion criteria included being currently employed as a mental health worker in Timor Leste and consenting to participate in the study. English versions of all scales were used. The rationale for using English scales was because all respondents were both the NGO workers and the Ministry of Health workers from the main referral hospital were fluent in written English. We were not able to do formal English language assessments on them due to time constraints, however, we prepared a Bahasa Indonesia translation, which was forward and back translated using WHO translation guidelines, in the event it was required for any respondents. Unfortunately, it was not possible to prepare formal validations in the national vernacular language of Tetum due to time and logistic constraints.

### Measures

1. *Mindfulness*: Mindfulness was assessed using the Toronto Mindfulness Scale (TMS), a widely used instrument that measures the levels of mindfulness in individuals. The TMS consists of two subscales: De-centering and Curiosity, which together capture the essence of mindfulness as a state of being aware and attentive to the present moment without judgment. This scale has demonstrated strong psychometric properties and is particularly relevant in understanding how mindfulness relates to psychological well-being in diverse populations [16].
2. *Cognitive Fusion*: Cognitive fusion was measured using the Cognitive Fusion Questionnaire (CFQ), which evaluates the extent to which individuals become entangled with their thoughts and treat them as literal truths. High levels of cognitive fusion are associated with greater psychological distress, as individuals struggle to separate themselves from their internal experiences. The CFQ is a validated tool that provides insights into how cognitive fusion contributes to mental health outcomes [7, 8].
3. *Coping Strategies*: Coping strategies were evaluated using the DBT-Ways of Coping Checklist (DBT-WCCL). This checklist includes subscales for both adaptive and maladaptive coping strategies. Adaptive strategies, such as problem-solving and seeking social support, are associated with better psychological outcomes, while maladaptive strategies, like avoidance and self-blame, can exacerbate distress. The DBT-WCCL is particularly useful in clinical settings to understand how individuals manage stress and adversity [20].
4. *Depression, Anxiety, and Stress*: The Depression Anxiety Stress Scales (DASS-21) were used to measure symptoms of depression, anxiety, and stress. This 21-item scale provides a comprehensive assessment of these three negative emotional states, with subscales for each dimension. The DASS-21 is a reliable and valid measure that has been extensively used in both clinical and non-clinical populations to assess the severity of these symptoms [19].

### Procedure

Thirty-seven participants (*n* = 37) were recruited through professional networks and organizational contacts, from two separate visits to Timor Leste in 2024 to train mental health workers in both the national mental health NGO, PRADET, and also the national referral hospital. They were provided with an information sheet explaining the study's purpose, procedures, and confidentiality assurances. Informed consent was obtained from all participants before they completed the questionnaires. The surveys were administered electronically, and participants were encouraged to complete them in a quiet and private setting.

### Data analysis

Data were analysed using descriptive statistics to summarize the demographic characteristics of the sample and the main study variables. Pearson correlation coefficients were calculated to examine the relationships between mindfulness, cognitive fusion, coping strategies, and psychological distress. Multiple regression analyses were conducted to explore the predictive value of mindfulness, cognitive fusion, and coping strategies on depression, anxiety, and stress levels. Statistical significance was set at *p* < 0.05.

## Results

Table 1 presents the demographic characteristics of the respondents with an average age of 38 years and a standard deviation of 9 years, indicating a diverse age distribution. The thirty seven respondents are categorized into three age groups: 27.0% are 30 years old or younger, 29.7% fall within the 31–40 age range, and the largest group, comprising 43.2%, is over 40 years old. This suggests a predominance of older individuals in the sample.

**Table 1:** Demographic characteristics

	Mean	Standard Deviation	n	%
Age (year)	38	9		
Age category				
≤ 30 years old			10	27.0%
31–40 years old			11	29.7%
> 40 years old			16	43.2%

The reliability analysis indicates that all instruments used in the study demonstrate acceptable internal consistency with Cronbach’s alpha values ranging from 0.688 to 0.919 (see Table 2).

**Table 2:** Reliability analysis

Instrument	No. of items	Cronbach’s α
Depression	7	0.919
Anxiety	7	0.908
Stress	7	0.882
Mindfulness (Curiosity)	6	0.692
Mindfulness (De-Centering)	7	0.789
Cognitive Fusion	7	0.902
Coping strategies (skills use)	38	0.881
Coping strategies (General dysfunctional coping)	15	0.810
Coping strategies (Blaming others)	6	0.688
Psychological Flexibility	7	0.863

The Shapiro–Wilk test results indicate that most variables deviate significantly from a normal distribution with *p*-values less than 0.05 (see Table 3) Specifically, Depression (*p* < 0.001), Anxiety (*p* = 0.001), Stress (*p* = 0.041), Mindfulness (Curiosity) (*p* = 0.002), Mindfulness (De-Centering) (*p* = 0.050), and General Dysfunctional Coping (*p* = 0.007) all exhibit significant deviations from normality. However, Cognitive Fusion (*p* = 0.373), Coping Strategies—Skills Use (*p* = 0.131), Blaming Others (*p* = 0.356), and Psychological Flexibility (*p* = 0.187) do not significantly differ from a normal distribution indicating acceptable normality. In terms of skewness and kurtosis, most values fall within the ± 2 range, suggesting moderate departures from normality.

**Table 3:** Normality test

	Mean	Standard Deviation	Skewness	Kurtosis	Shapiro–Wilk	*p*-value of Shapiro–Wilk
Depression	9.351	11.285	1.335	1.088	0.812	< 0.001
Anxiety	14.432	12.633	0.779	− 0.574	0.888	0.001
Stress	14.973	11.388	0.363	− 0.753	0.938	0.041
Mindfulness (Curiosity)	18.919	4.418	− 0.274	− 1.167	0.89	0.002
Mindfulness (De-Centering)	19.054	6.561	− 0.269	− 1.061	0.941	0.05
Cognitive Fusion	22.541	10.262	0.511	− 0.019	0.969	0.373
Coping strategies (skills use)	2.231	0.354	− 0.328	− 0.931	0.954	0.131
Coping strategies (general dysfunctional coping)	1.845	0.508	− 0.699	− 0.51	0.914	0.007
Coping strategies (blaming others)	1.523	0.606	0.028	− 0.675	0.968	0.356
Psychological Flexibility	19.703	8.8	0.561	0.166	0.959	0.187

Table 4 outlines the severity of psychological distress among the respondents, highlighting three specific areas: depression, anxiety, and stress. For depression, 64.9% of the respondents are categorized as having normal severity, while a smaller percentage experience mild (2.7%), moderate (18.9%), severe (2.7%), and extremely severe (10.8%) symptoms. In terms of anxiety, 37.8% are considered normal, with 5.4% experiencing mild, 18.9% moderate, 10.8% severe, and a notable 27.0% classified as extremely severe. Lastly, regarding stress anxiety, 56.8% are normal, while 10.8% show mild, 5.4% moderate, 21.6% severe, and 5.4% extremely severe symptoms. Overall, the data indicates that while many respondents experience normal levels of distress, a significant portion, especially regarding anxiety, exhibits moderate to extreme levels of distress.

**Table 4:** Severity of psychological distress

	Normal		Mild		Moderate		Severe		Extremely severe	
	*n*	%	*n*	%	*n*	%	*n*	%	*n*	%
Depression severity	24	64.9%	1	2.7%	7	18.9%	1	2.7%	4	10.8%
Anxiety severity	14	37.8%	2	5.4%	7	18.9%	4	10.8%	10	27.0%
Stress severity	21	56.8%	4	10.8%	2	5.4%	8	21.6%	2	5.4%

Since the data did not meet the normality assumption, Spearman’s rank-order correlation was used to examine the relationships among the study variables (see Table 5). Significant positive correlations were found between Depression and Anxiety (Spearman’s rho = 0.649, *p* < 0.001), and between Depression and Stress (Spearman’s rho = 0.753, *p* < 0.001), indicating strong associations among these constructs. Depression was also significantly correlated with Cognitive Fusion (Spearman’s rho = 0.445,* p* = 0.006) and Blaming Others (Spearman’s rho = 0.422, *p* = 0.009), and negatively correlated with Coping Strategies (Skills Use) (Spearman’s rho =–0.341, *p* = 0.039). Anxiety and Stress were highly correlated (Spearman’s rho = 0.855, *p* < 0.001), and both were significantly associated with Cognitive Fusion, General Dysfunctional Coping, and Blaming Others. Mindfulness (De-Centering) showed a strong positive correlation with Mindfulness (Curiosity) (Spearman’s rho = 0.770, *p* < 0.001), and was also weakly associated with General Dysfunctional Coping (Spearman’s rho = 0.343, *p* = 0.038). Overall, the results suggest that higher levels of depression, anxiety, and stress are linked to greater cognitive fusion and dysfunctional coping, while effective coping skills are negatively associated with depression.

**Table 5:** Relationships between mindfulness, cognitive fusion, coping strategies, and psychological distress

Variable		Depression	Anxiety	Stress	Mindfulness Curiosity	Mindfulness De-Centering	Cognitive Fusion	Coping strategies (skills use)	Coping strategies (general dysfunctional coping)	Coping strategies (blaming others)
Depression	Spearman's rho	–								
	*p*-value	–								
Anxiety	Spearman's rho	0.649***	–							
	*p*-value	< 0.001	–							
Stress	Spearman's rho	0.753***	0.855***	–						
	*p*-value	< 0.001	< 0.001	–						
Mindfulness Curiosity	Spearman's rho	0.026	0.108	0.073	–					
	*p*-value	0.88	0.526	0.67	–					
Mindfulness De-Centering	Spearman's rho	0.175	0.215	0.183	0.77***	–				
	*p*-value	0.299	0.202	0.278	< 0.001	–				
Cognitive Fusion	Spearman's rho	0.445**	0.586***	0.581***	0.172	0.275	–			
	*p*-value	0.006	< 0.001	< 0.001	0.308	0.1	–			
Coping strategies (skills use)	Spearman's rho	− 0.341*	0.106	0.019	0.161	0.034	0.2	–		
	*p*-value	0.039	0.531	0.91	0.342	0.843	0.234	–		
Coping strategies (general dysfunctional coping)	Spearman's rho	0.297	0.537***	0.434**	0.248	0.343*	0.458**	0.605***	–	
	*p*-value	0.075	< 0.001	0.007	0.139	0.038	0.004	< 0.001	–	
Coping strategies (blaming others)	Spearman's rho	0.422**	0.606***	0.538***	0.099	0.238	0.321	0.365*	0.801***	–
	*p*-value	0.009	< 0.001	< 0.001	0.56	0.156	0.052	0.026	< 0.001	–

*p < 0.05, ** *p* < 0.01, *** *p* < 0.001

Table 6 presents the results of multiple regression analyses assessing the predictive roles of mindfulness, cognitive fusion, coping strategies, and BRT on levels of depression, anxiety, and stress. Notable findings include that cognitive fusion significantly predicts increased levels of depression (β = 0.418, *p* < 0.05), anxiety (β = 0.491, *p* < 0.05), and stress (β = 0.482, *p* < 0.05). Coping strategies that involve skills use are linked to a significant reduction in depression (β = -19.697, *p* < 0.05), although they do not significantly affect anxiety or stress. The coping strategy of blaming others significantly predicts higher anxiety (β = 12.527, *p* < 0.05) and stress (β = 9.055, *p* < 0.05). Mindfulness factors show minimal predictive value across outcomes. The models explain approximately 52% to 56% of the variance in depression, anxiety, and stress levels, with all models demonstrating statistical significance (*F* values > 4.4). Residual normality assumptions were met for all models, as indicated by non-significant Shapiro–Wilk tests (*p* > 0.05). Overall, cognitive fusion and specific coping strategies are critical in understanding mental health outcomes.

**Table 6:** Multiple regression analyses to explore the predictive value of mindfulness, cognitive fusion, coping strategies, and BRT on depression, anxiety, and stress levels

Model	Depression	Anxiety	Stress
	Unstandardized coefficient (*t* value)	Unstandardized coefficient (*t* value)	Unstandardized coefficient (*t* value)
Intercept	26.462 (2.581*)	− 4.436 (− 0.375)	6.388 (0.591)
Mindfulness (Curiosity)	− 0.213 (− 0.425)	0.321 (0.553)	− 0.061 (− 0.115)
Mindfulness (De-Centering)	0.259 (0.739)	− 0.021 (− 0.052)	0.053 (0.144)
Cognitive Fusion	0.418 (2.257*)	0.491 (2.298*)	0.482 (2.465*)
Coping strategies (skills use)	− 19.697 (− 3.568*)	− 5.828 (− 0.914)	− 7.074 (− 1.214)
Coping strategies (General dysfunctional coping)	7.266 (1.101)	− 0.746 (− 0.098)	− 0.452 (− 0.065)
Coping strategies (Blaming others)	3.468 (0.836)	12.527 (2.615*)	9.055 (2.070*)
Psychological Flexibility	− 0.110 (− 0.501)	− 0.130 (− 0.516)	0.035 (0.154)
*R*^2^	0.558	0.529	0.517
*F* value	5.226*	4.659*	4.428*
Normality of the residuals (Shapiro–Wilk)	0.950 (*p* = 0.096)	0.972 (*p* = 0.465)	0.982 (*p* = 0.802)

*p < 0.05

## Discussion

The present study sought to examine the relationships between mindfulness, cognitive fusion, coping strategies, and psychological distress among mental health workers in Timor Leste. The findings revealed significant associations between these variables, offering valuable insights into the psychological mechanisms underlying mental health in this population.

The strong positive correlation observed between the two mindfulness facets—curiosity and de-centering—suggests these components are closely related and often co-occur as part of an overarching mindful awareness. Interestingly, both mindfulness facets also showed positive correlations with cognitive fusion, a finding that diverges from the typical assumption that greater mindfulness is associated with lower fusion. This may reflect the early stages of mindfulness development in high-stress contexts such as post-conflict Timor Leste, where increased awareness could initially amplify contact with distressing internal experiences before individuals develop the defusion skills needed to respond flexibly [24].

Despite this complex relationship, cognitive fusion was clearly associated with higher levels of psychological distress, particularly anxiety, stress, and depression as well as dysfunctional coping strategies. These findings are consistent with theoretical models of Acceptance and Commitment Therapy, which posit that cognitive fusion intensifies emotional suffering by increasing the rigidity of thought patterns and reducing the capacity for adaptive regulation [7, 8, 11, 12]. Hence, fused thinking can lead individuals to engage in maladaptive coping behaviours aimed at controlling or avoiding unwanted internal experiences. Thus, while mindfulness and cognitive fusion may coexist in some contexts, reducing fusion through mindfulness-based strategies remains a critical therapeutic goal to mitigate distress and improve coping among mental health workers in this setting.

Coping strategies also played a significant role in predicting psychological distress. Specifically, skills use was associated with lower levels of depression, highlighting the importance of adaptive coping mechanisms in mitigating mental health issues [17]. Conversely, dysfunctional coping strategies, such as blaming others, were linked to increased anxiety and stress. The lack of a significant relationship between mindfulness—both curiosity and de-centering—and psychological distress (depression, anxiety, and stress) warrants further reflection, particularly in light of cultural factors. In Timor Leste, cultural norms that emphasize collectivism, modesty, and deference to authority may shape how individuals experience and express internal states such as curiosity or self-reflection. The curiosity dimension of mindfulness, which involves open and inquisitive engagement with one’s thoughts and emotions, may not be as culturally encouraged or readily expressed in contexts where emotional restraint or spiritual explanations for suffering are more normative. As such, higher levels of self-reported curiosity might not translate into meaningful psychological benefits without corresponding cultural or social support for reflective practices. Furthermore, mindfulness as conceptualized in Western psychological frameworks may not fully align with local understandings of mental well-being or traditional coping methods, potentially limiting its perceived relevance or utility. These cultural nuances highlight the need for culturally adapted mindfulness frameworks that resonate with local values and lived experiences.

These results underscore the need for interventions aimed at promoting adaptive coping skills among mental health workers to enhance their resilience and well-being [4]. The significant negative association between the use of coping skills and depression, as shown in the regression analysis, highlights the protective role of adaptive coping strategies in mitigating psychological distress. In contrast, dysfunctional coping strategies—particularly blaming others—were significantly associated with higher levels of anxiety and stress, supporting prior research that links such strategies with increased emotional dysregulation and poorer mental health outcomes. These findings underscore the importance of fostering psychological flexibility and adaptive coping strategies in mental health interventions for mental health workers in Timor Leste, particularly given the lingering impact of trauma and the demands of working in a resource-limited, post-conflict context.

These findings reinforce the importance of fostering adaptive coping mechanisms—such as problem-solving, emotional regulation, and mindful acceptance—especially within high-risk professional groups like mental health workers in post-conflict settings such as Timor Leste.

It is noteworthy to remember that psychological constructs explored in this study may be influenced by demographic and experiential differences among mental health workers in Timor Leste. For instance, variations in age and years of work experience can shape how individuals engage with mindfulness, coping strategies, and psychological flexibility [27]. Younger or less experienced mental health workers may rely more heavily on dysfunctional coping strategies or experience higher cognitive fusion due to limited exposure to structured supervision, support systems, or opportunities for reflective practice. In contrast, those with more years in the field might have developed more adaptive coping mechanisms and greater psychological flexibility, contributing to lower distress levels. Additionally, the motivation behind choosing to become a mental health worker—whether driven by personal values, community service ideals, or economic necessity—can also affect how these individuals relate to psychological distress and coping mechanisms [10]. Mental health workers who identify strongly with their role as a calling may demonstrate greater resilience, while those facing role strain or misalignment with expectations may be more vulnerable to distress.

Cultural context also plays a vital role in shaping the psychological functioning of mental health workers in Timor Leste. The strong communal and familial ties prevalent in Timorese society may influence the way distress is perceived and expressed, as well as the kinds of coping strategies deemed acceptable. For example, blaming others—a significant predictor of anxiety and stress in this study—might be partially shaped by cultural norms around collectivism and accountability [9]. Moreover, the internalisation of cultural values such as deference to authority, community harmony, and spiritual belief systems may impact cognitive fusion, especially when individuals experience tension between traditional expectations and professional responsibilities [23]. These cultural underpinnings highlight the importance of culturally adapted interventions that not only promote individual psychological skills like mindfulness and psychological flexibility, but also take into account the socio-cultural realities that shape how mental health workers in Timor Leste experience and manage stress.

Despite these important findings, the study has several limitations. Firstly, the cross-sectional design precludes causal inferences, limiting our ability to determine the directionality of the relationships between the variables. Longitudinal studies are needed to establish causality and examine the stability of these relationships over time. Secondly, the reliance on self-report measures may introduce bias, as participants might underreport or overreport their symptoms and coping strategies due to social desirability or recall biases [25]. Future research could benefit from incorporating objective measures and multi-method approaches to validate the findings. Cultural and linguistic differences may influence how items are interpreted, potentially affecting the accuracy of the results. Future research is recommended to conduct thorough psychometric evaluations, specifically with samples from Timor Leste to ensure the measurements are appropriate and meaningful for this population.

Additionally, the lack of comprehensive demographic data—such as work setting, years of experience, and personal motivations for entering the profession—limited the possibility of conducting subgroup or moderator analyses that could provide more nuanced insights. For example, differences in psychological flexibility, coping patterns, or distress levels across different age groups or experience levels could not be explored. This restricts a more contextualised understanding of how these psychological processes manifest in diverse profiles of mental health workers. Lastly, the relatively small sample size may not be representative of all mental health professionals in Timor Leste, which limits the generalizability of the results. Future studies should aim to recruit larger and more demographically diverse samples to improve external validity and enable deeper analysis of individual and contextual factors.

## Conclusion

In conclusion, this study highlights the critical roles of cognitive fusion and coping strategies in predicting psychological distress among mental health workers in Timor Leste. The findings suggest that interventions targeting cognitive fusion and promoting adaptive coping skills could be beneficial in reducing depression, anxiety, and stress in this population. Additionally, while mindfulness was associated with cognitive fusion, its direct impact on psychological distress was minimal in this study, indicating the need for further research to explore its complex role in mental health.

Addressing the mental health needs of mental health workers is essential, given their critical role in supporting the well-being of the broader population. By fostering a better understanding of the psychological mechanisms at play, we can develop more effective interventions to support these professionals and enhance their resilience in the face of ongoing challenges.

## Data Availability

The datasets generated and/or analysed during the current study are available from the corresponding author on reasonable request. The authors confirm that their submitted work is original, has not been published elsewhere. This study was reviewed and approved by the Universiti Malaysia Sabah Medical Research Ethics Committee, with the approval reference number: JKEtika 4/24 (3).
